# The incidence of varicella and herpes zoster in Massachusetts as measured by the Behavioral Risk Factor Surveillance System (BRFSS) during a period of increasing varicella vaccine coverage, 1998–2003

**DOI:** 10.1186/1471-2458-5-68

**Published:** 2005-06-16

**Authors:** W Katherine Yih, Daniel R Brooks, Susan M Lett, Aisha O Jumaan, Zi Zhang, Karen M Clements, Jane F Seward

**Affiliations:** 1 Department of Ambulatory Care and Prevention, Harvard Medical School and Harvard Pilgrim Health Care, Boston, USA; 2 Department of Epidemiology, Boston University School of Public Health, Boston, USA; 3 Division of Epidemiology and Immunization, Bureau of Communicable Disease Control, Massachusetts Department of Public Health, Boston, USA; 4 Health Investigation Branch, Division of Health Studies, Agency for Toxic Substance and Disease Registry, Centers for Disease Control and Prevention, Atlanta, USA; 5 Health Survey Program; Center for Health Information, Statistics, Research and Evaluation; Massachusetts Department of Public Health; Boston, USA; 6 Applied Statistics, Evaluation and Technical Services; Bureau of Family and Community Health; Massachusetts Department of Public Health; Boston, USA; 7 Viral Vaccine-Preventable Disease Branch, Epidemiology and Surveillance Division, National Immunization Program, Centers for Disease Control and Prevention, Atlanta, USA

## Abstract

**Background:**

The authors sought to monitor the impact of widespread varicella vaccination on the epidemiology of varicella and herpes zoster. While varicella incidence would be expected to decrease, mathematical models predict an initial increase in herpes zoster incidence if re-exposure to varicella protects against reactivation of the varicella zoster virus.

**Methods:**

In 1998–2003, as varicella vaccine uptake increased, incidence of varicella and herpes zoster in Massachusetts was monitored using the random-digit-dial Behavioral Risk Factor Surveillance System.

**Results:**

Between 1998 and 2003, varicella incidence declined from 16.5/1,000 to 3.5/1,000 (79%) overall with ≥66% decreases for all age groups except adults (27% decrease). Age-standardized estimates of overall herpes zoster occurrence increased from 2.77/1,000 to 5.25/1,000 (90%) in the period 1999–2003, and the trend in both crude and adjusted rates was highly significant (p < 0.001). Annual age-specific rates were somewhat unstable, but all increased, and the trend was significant for the 25–44 year and 65+ year age groups.

**Conclusion:**

As varicella vaccine coverage in children increased, the incidence of varicella decreased and the occurrence of herpes zoster increased. If the observed increase in herpes zoster incidence is real, widespread vaccination of children is only one of several possible explanations. Further studies are needed to understand secular trends in herpes zoster before and after use of varicella vaccine in the United States and other countries.

## Background

Prior to the licensure of varicella vaccine in the U.S. in 1995, varicella (chickenpox) was a highly prevalent disease of childhood, resulting in approximately four million cases and an average of 10,630–13,500 hospitalizations and 90 deaths in the U.S. each year [1-4]. After primary infection, the varicella zoster virus (VZV) lies dormant in sensory ganglia and can reactivate later, producing herpes zoster (shingles) and sometimes persistent, debilitating pain and other serious complications [5]. Associated with a decline in specific cell-mediated immunity, herpes zoster affects some 600,000–900,000 mostly elderly or immunocompromised people in the U.S. each year, its incidence increasing with age [1,6].

Establishing and/or enhancing varicella and herpes zoster surveillance has been essential in order to assess the impact of widespread varicella vaccination [7,8]. Despite the fact that national vaccination coverage among children 19–35 months of age had risen to 85 percent by 2003 [9], varicella did not become a nationally notifiable disease in the U.S. until that year. Herpes zoster has never been a nationally notifiable disease, and no states require reporting of cases. Studies of herpes zoster using different methods have reported unadjusted incidence rates ranging from 1.3 to 4.8 per 1,000 population [10-13], but there is little documentation of secular trends over time. Some studies suggest that re-exposure to VZV after primary infection may decrease the risk of herpes zoster through immunologic boosting [14-16]. If this is the case, widespread use of varicella vaccine could, by reducing circulating VZV, increase the incidence of herpes zoster over the first few decades of universal childhood vaccination. On the other hand, studies in leukemic as well as healthy children have found a lower incidence and severity of herpes zoster among vaccinated than among comparable unvaccinated children [17-21], which would appear to bode well for the more distant future if vaccine coverage reaches high levels. Clearly, consistent and long-term surveillance for herpes zoster will be necessary in order to fully assess the impact of varicella vaccination on the epidemiology of zoster.

Starting in 1998, as uptake of varicella vaccine was increasing (due in part to immunization requirements for daycare and school entry), the Massachusetts Department of Public Health, in collaboration with the Centers for Disease Control and Prevention (CDC), enhanced varicella and herpes zoster surveillance by including questions in the Behavioral Risk Factor Surveillance System (BRFSS) about the occurrence of these diseases. Researchers in Kentucky had used both the BRFSS and a school-based cohort study to estimate age-specific varicella incidence in 1990–1992 and found generally close concordance between results of the two methods [22].

Herein, we analyze varicella and herpes zoster incidence as estimated by the Massachusetts BRFSS in the period 1998–2003.

## Methods

### Vaccine use and coverage

The Massachusetts Department of Public Health began to distribute varicella vaccine in September 1996 for use in 12–18-month-olds and susceptible sixth-graders. In October 1997, eligibility was expanded to include all children between 1 and 18 years of age. Beginning in August 1998, proof of immunity to varicella (written documentation of age-appropriate immunization or physician-certified reliable history of disease) was required for child-care center attendance for children ≥ 19 months of age who were born after 1996. Finally, in September 1999, proof of immunity became required for entry into kindergarten and seventh grade.

Vaccine coverage estimates were obtained from the National Immunization Survey. The CDC conducts this random-digit-dial telephone survey of households regarding the immunization status of children 19–35 months of age each year. National Immunization Survey methods have been published elsewhere [23].

### Varicella and herpes zoster incidence

The BRFSS is an ongoing, random-digit-dial telephone survey of adults aged 18 years and older and gathers information on health characteristics, risks, and preventive behaviors. The survey is conducted in all U.S. states in collaboration with the CDC and state health departments. Once a household is contacted, one adult is randomly selected for interview. Characteristics of the BRFSS are described in more detail elsewhere [24].

The BRFSS includes a core set of questions asked by all states, as well as optional topics added by individual states. Massachusetts added questions about chickenpox and shingles among all household members in 1998–2000 and 2002–2003. Respondents were first asked to enumerate all household members and their ages. Trained interviewers then asked respondents whether any household members had chickenpox in the past 12 months and the current age(s) of the affected household member(s). Respondents were also asked whether any household members had *ever *had shingles, the current age(s) of affected household member(s), and the age(s) when they had shingles.

For these analyses, we calculated the annual age-specific incidence of varicella and herpes zoster per 1,000 population. In 1998 only, the response to the age-at-shingles question was recorded only in five-year age groups (0–4 years, 5–9 years, etc.), so annual incidence of herpes zoster could not be calculated for that year. We made some simplifying assumptions in estimating age-specific annual incidence of varicella and herpes zoster. For varicella, each case that was reported as occurring in the previous 12 months was assigned to the individual's current age. For herpes zoster, a case was counted as occurring within the previous year if the age at the time of the reported case was the same as or 1 year less than the current age. Our data on herpes zoster combine incident and recurrent cases, as no distinction was made between these in the interview. In presenting the varicella data, we used five standard child/adolescent age groups and grouped adults 20 years and older into a sixth. In the case of herpes zoster, we used four 20–25-year age intervals in order to increase the stability of the data.

BRFSS data were weighted to account for probability of selection and differential participation by age, sex, and race/ethnicity. All data were analyzed using appropriate procedures in SAS and SUDAAN, taking into account the survey sampling scheme, intra-class correlation among household members, and weighting of the data (SAS Institute, Cary, NC; SUDAAN, RTI, Research Triangle Park, NC). Tests for linear trend were done using logistic regression (with incidence as the outcome and year as the exposure variable), adjusting for age in comparisons of overall incidence. Age standardization was based on the standard 2000 U.S. population [25] in 14 mostly five-year age groups: 1–14, 15–19, 20–24, 25–29, 30–34, 35–39, 40–44, 45–49, 50–54, 55–59, 60–64, 65–69, 70–74, and 75+. The oldest group was not further subdivided due to diminishing cell sizes.

## Results

### Response rate

The response rate for the Massachusetts BRFSS as a whole was 59% in 1998, 55% in 1999, 41% in 2000, 66% in 2002, and 65% in 2003. The large apparent increase in response rate in 2002 was mainly due to the fact that in that year BRFSS expanded the number of case disposition codes in order to more accurately differentiate between different types of response and non-response and to bring the BRFSS disposition codes more in line with those recommended by the American Association for Public Opinion Research (AAPOR). This broader array of codes allows for more accurate accounting of participation rates, such as response rates (pers. comm., Dr. Michael Link, BRFSS, CDC). The number of households responding to the varicella-herpes zoster module ranged between 4,200 and 4,900, representing between 11,000 and 14,000 members.

### Vaccine coverage

**Figure 1:**
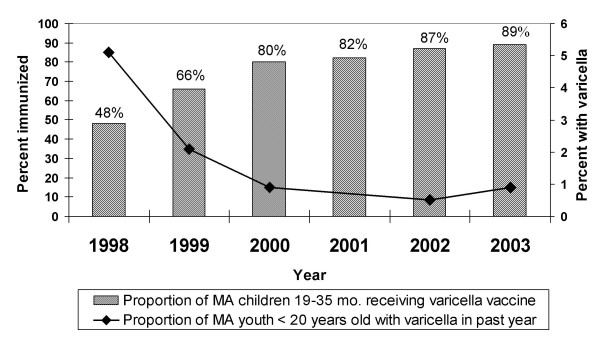
Varicella immunization levels and proportion of Massachusetts youth with varicella, 1998–2003. Annual National Immunization Survey estimates of varicella immunization levels and percent of Massachusetts residents < 20 years of age with varicella in the past year by date of interview, 1998–2003.

Using National Immunization Survey estimates for Massachusetts children 19–35 months of age [26], coverage increased from 23 percent in 1997 to 48 percent in 1998 and reached 89 percent in 2003 (Figure 1).

### Varicella incidence

In 1998, the highest incidences of varicella were observed in children 1–4 years of age (83 per 1,000) and 5–9 years of age (76 per 1,000). Between 1998 and 2003, overall unadjusted varicella incidence declined by 13.0 cases per 1,000 (79%), from 16.5 to 3.5 per 1,000 (p < 0.0001; Table 1 and Figure 1). The overall incidence of varicella was higher in 2003 than in 2002 but not statistically significantly so. Over 1998–2003, incidence declined for all age groups, with the greatest decreases among infants, 1–4-, 5–9-, and 15–19-year olds (100%, 89%, 80%, and 92%, respectively) and the smallest among adults (27%). The trends were highly significant (p < 0.0001) for the 1–4- and 5–9-year olds and significant (p = 0.02) for the 10–14-year olds.

**Table 1:** Age-specific incidence (cases per thousand) of varicella and trends, Massachusetts, 1998–2003

Year	Parameter	Age group in years						
		
		< 1	1–4	5–9	10–14	15–19	20+	All ages, crude
1998								
	n^a^	144	762	1025	922	791	8241	11885
	Cases	4	66	83	23	9	34	219
	Incidence	15.2	82.6	76.4	18.6	30.2	2.2	16.5
	95% CI	4.7–49.5	59.9–113.9	55.0–104.5	10.8–32.0	8.3–109.4	1.4–3.5	12.8–21.3
								
1999								
	n^a^	318	1603	1131	1094	898	8483	13527
	Cases	4	46	41	11	3	17	122
	Incidence	6.2	36.5	38.2	10.4	2.9	1.7	10.9
	95% CI	1.2–31.6	21.1–63.0	24.8–59.0	4.8–22.5	0.6–13.4	0.9–3.4	7.8–15.1
								
2000								
	n^a^	139	671	790	918	693	7518	10729
	Cases	1	7	9	9	3	3	32
	Incidence	11.4	11.6	10.3	8.8	4.2	0.3	2.7
	95% CI	1.4–53.7	4.7–25.6	4.8–20.6	4.0–18.1	0.8–19.8	0.1–1.23	1.8–4.3
								
2002								
	n^a^	161	600	780	831	705	7789	10866
	Cases	1	4	5	3	1	6	20
	Incidence	8.7	4.3	8.0	4.6	1.4	0.5	1.7
	95% CI	1.1–44.6	1.2–13.9	3.0–20.0	1.3–15.4	0.2–9.7	0.2–1.1	1.0–2.8
								
2003								
	n^a^	155	613	827	914	726	8012	11247
	Cases	0	7	15	6	2	10	40
	Incidence	0.0	8.8	15.0	6.4	2.3	1.6	3.5
	95% CI		3.6–20.2	7.8–27.0	2.7–14.4	0.6–9.2	0.8–3.1	2.3–5.2
								
1998–2003								
	% change for period	-100%	-89%	-80%	-66%	-92%	-27%	-79%
	p for trend test	n.a.	<0.0001	<0.0001	0.02	0.15	0.16	<0.0001

^a ^N's exclude individuals for whom age or varicella status was missing. The number excluded for these reasons was 494, 233, 478, and 195 in 1999, 2000, 2002, and 2003, respectively. Values for n's and numbers of cases are unweighted, whereas incidence rates and 95% confidence intervals are based on weighted analysis.

### Herpes zoster incidence

**Figure 2:**
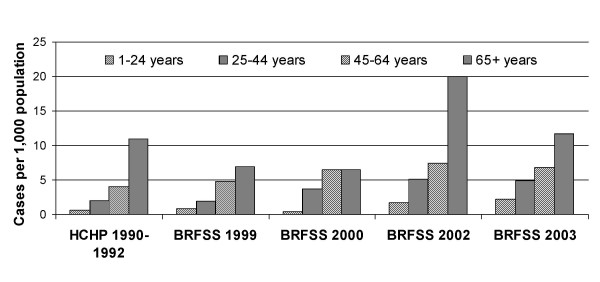
Annual incidence of herpes zoster by age, 1999–2003. Annual incidence of herpes zoster by age: comparison of Harvard Community Health Plan (HCHP), 1990–1992 [6], and the Massachusetts Behavioral Risk Factor Surveillance System (BRFSS), 1999, 2000, 2002, and 2003.

Overall age-standardized (and crude) incidence per 1,000 of herpes zoster was 2.77 (2.23) in 1999, 3.54 (3.57) in 2000, 6.47 (6.51) in 2002, and 5.25 (5.38) in 2003 (Table 2), and the increasing trend was highly significant for both the age-adjusted (p < 0.0009) and crude (p < 0.0001) incidences. The increase in the age-standardized incidence was 90 percent over the five-year period. Over this period, age-specific incidences increased by 41–161 percent, although the annual age-specific incidence estimates were somewhat unstable (Table 2, Figure 2).

**Table 2:** Age-specific incidence (cases per thousand) of herpes zoster and trends, Massachusetts, 1999–2003

Year	Parameter	Age group in years					
		
		1–24	25–44	45–64	65+	All ages, crude	All ages, standardized ^a^
1999							
	n^b^	5655	4230	2214	911	13010	
	Cases	5	14	11	10	40	
	Incidence	0.87	1.87	4.82	6.9	2.23	2.77
	95% CI	0.25–3.05	0.90–3.88	2.36–9.86	2.97–16.05	1.46–3.43	1.82–4.20
							
2000							
	n^b^	3668	3449	2211	1121	10449	
	Cases	2	12	13	8	35	
	Incidence	0.46	3.74	6.45	6.45	3.57	3.54
	95% CI	0.11–1.91	2.04–6.76	3.60–11.29	3.04–13.16	2.50–5.08	2.48–5.03
							
2002							
	n^b^	3469	3234	2451	1303	10457	
	Cases	6	14	20	23	63	
	Incidence	1.70	5.13	7.42	20.03	6.51	6.47
	95% CI	0.71–4.03	2.65–9.70	4.39–12.28	12.46–30.59	4.86–8.67	4.92–8.47
							
2003							
	n^b^	3698	3260	2700	1278	10936	
	Cases	8	16	18	14	56	
	Incidence	2.19	4.88	6.79	11.74	5.38	5.25
	95% CI	1.05–4.50	2.86–8.20	4.02–11.23	6.57–20.12	4.03–7.14	3.96–6.94
							
1999–2003							
	% change for period	152%	161%	41%	70%	141%	90%
	p for trend test	0.10	0.03	0.42	0.03	0.0001	0.0009

^a ^Incidence is standardized using the standard 2000 U.S. population. The p-value is from age-adjusted analysis for trend using logistic regression.

^b ^N's exclude individuals for whom age or herpes zoster status was missing. The number excluded for these reasons was 1011, 513, 887, and 506 in 1999, 2000, 2002, and 2003, respectively. Values for n's and numbers of cases are unweighted, whereas incidence rates and 95% confidence intervals are based on weighted analysis.

## Discussion

The age-specific incidences of varicella in 1998 were generally within the range of estimates reported in studies conducted using a variety of methods in other parts of the U.S. prior to the introduction of the vaccine [1,22,27-29]. We noted a marked reduction in varicella incidence in all age groups in Massachusetts over the period 1998–2003; the decline was between 66 and 100 percent for all groups except adults, in whom incidence decreased by 27 percent. Vaccine coverage in Massachusetts 19–35-month-olds increased from 48 to 89 percent over the same 6-year period.

Seward et al. [30] observed a similarly consistent decline over the 6-year period of 1995–2000 in three areas of active surveillance, whose range of vaccine coverage as measured by the National Immunization Survey encompassed Massachusetts' coverage each year. Likewise, Jumaan et al. [31] documented a decline in age-adjusted incidence of varicella from 2.63 per 1,000 in 1995 to 0.92 per 1,000 in 2002. Decreases in varicella incidence after vaccine licensure have been noted in Illinois, Michigan, Texas, and West Virginia as well [32]. Similarly, large decreases in incidence among children in childcare centers and among U.S. Navy recruits have been associated with varicella vaccination [33,34].

The overall incidence of varicella was higher in 2003 than in 2002, although the difference was not statistically significant. No such upturn was apparent in Massachusetts Department of Public Health surveillance data, which showed an overall decrease of 20 percent in cases and incidence between 2002 and 2003, in spite of increased efforts to stimulate varicella reporting. (Overall incidence of herpes zoster decreased from about 6 to about 5 per 1,000 from 2002 to 2003, but the change was not statistically significant for either the crude or the age-standardized estimates.)

Age-standardized overall annual herpes zoster incidence increased by 2.5 cases per 1,000 (90%) over the 1999–2003 period, to 5.25 per 1,000, higher than the age-standardized estimates derived using the same standard population from a study in the Netherlands in 1994–1999 (3.34 per 1,000) [35] and from a study in Seattle, Washington in 1992–2002 (range: 3.47–4.05 per 1,000) [31] but not substantially higher than the estimate obtained from a study in France in 1997–1998 (4.55 per 1,000) [13]. The age-specific incidences of herpes zoster found here were consistently higher than in a Minnesota study of 50 years ago [12], and more than half of the age-specific point estimates in four other studies [11-13,6] fell below the 95 percent confidence intervals of our combined 2002–2003 estimates. (Confidence intervals for most of these previous studies were not available for comparison.) The four age groups showed increases of 41–161 percent, and the trend was significant for the 25–44 and 65+ age groups. In spite of the fact that the annual age-specific estimates of herpes zoster incidence were unstable, as can be seen particularly in the 65+ year-old age group (Figure 2), it does appear that there was a generally increasing trend in herpes zoster incidence for all age groups, based on these four years of data over a five-year period. This trend appears to conflict with findings from the Seattle study, a large longitudinal study in a health-maintenance organization in which age-adjusted as well as age-specific herpes zoster incidences remained relatively stable over the period 1992–2002, during the latter four years of which varicella incidence declined [31].

If in fact herpes zoster incidence is rising in the current period, there are several possible explanations, which may be operating in combination. One of these is varicella vaccination. Widespread varicella vaccination may end up increasing herpes zoster incidence among adults who have had varicella. Recent studies have suggested a protective effect against herpes zoster from exposure to varicella and/or children [14-16]. One mathematical model predicts a *long-term *(equilibrium state) elevated incidence of herpes zoster of 20 percent or more relative to pre-vaccination levels, under the assumption of immunologic boosting by exposure to VZV, if vaccine virus has a 75 percent or greater chance of reactivating to cause herpes zoster [36]. A later model by Brisson et al. [16], which was parameterized by means of a large, population-based survey and assumes that immunity from boosting lasts for 20 years, predicts a substantial increase in herpes zoster cases over the first 30–50 years after the initiation of mass vaccination, peaking about 20 years after the start of mass vaccination at an incidence of 51 percent over the pre-vaccination level and eventually falling below the initial incidence. Those aged 10–44 years at the start of mass vaccination will be most affected, according to the model, their lifetime risk exceeding 50 percent, compared to 33 percent in the pre-vaccination period – most will have been previously infected but will no longer experience boosting by exposure to children with varicella disease.

The Brisson model predicts the greatest rates of increase in herpes zoster incidence in the years just after the start of mass varicella vaccination, with a 40 percent increase over the pre-vaccination level expected by Year 10. Yet, over just a five-year period, we saw an increase of this magnitude in the 45–64-year age group and even larger increases in the other age groups, and it seems unlikely that varicella vaccination alone can explain our herpes zoster results. Other possible explanations include increases in the proportion of people with immunosuppressive conditions and therapies, in the duration of those conditions and treatments, and/or in the prevalence of other triggering factors. Some past studies have noted an increase in zoster incidence well before the availability of varicella vaccine, one finding 28- and 41-percent increases for Minnesota women and men, respectively, between the late 1940s and the late 1950s [12] and one finding a 35-percent increase between 1979 and 1997 in Canada [37]. Similarly, data from the National Health Interview Survey suggest that incidence of herpes zoster rose in the 60+ age groups between 1974 and 1994 [38].

Our study is subject to several limitations. Our method of estimating annual incidence of herpes zoster, by which a case was counted as occurring within the past year if the age at the time of the reported case was the same as *or 1 year less *than the age at interview, would tend to overestimate annual incidence because this encompasses a period greater than 12 months. The inclusion of recurrent cases with first-time cases would have the same effect. However, any overestimation due to these factors would be felt uniformly over age groups and time and would not be expected to influence year-to-year differences. Nevertheless, this would affect comparisons with other studies and likely contributes to our generally higher rates.

Secondly, varicella and herpes zoster incidences were based on respondent recall, both self- and proxy-report, and we did not validate the accuracy of the reporting. Proxy reporting may or may not introduce reporting error, depending on the health condition in question and the relationship of the respondent to the person about whom information is sought [39,40]. Regarding varicella, in our combined data for 1999 and 2000, a parent or step-parent was the respondent in more than 90 percent of households with children, and restricting the varicella analysis to these parent-respondents did not appreciably change our estimates of annual age-specific incidence. Researchers using a telephone survey to investigate varicella incidence in a city in Minnesota estimated through medical chart abstraction that 13 percent of parental reports of varicella in the previous year were either not varicella or occurred more than 13 months before the interview [28]. Our data could reflect similar over-reporting; however, error of this nature would not likely change from year to year and thus the trends would not be affected. One study found shingles self-report among the elderly to be accurate, with few false-positive and false-negative reports when compared to physician diagnosis [41]. We found no significant difference between shingles incidence in respondents of age 50 and older and shingles incidence in the same age group as reported by proxies.

Also, in this study, herpes zoster was not likely often diagnosed by laboratory testing. If the public or physicians became more aware of zoster in the latter years of the study, there might have been more of a tendency to attribute rash illness to zoster with each successive annual survey, leading to an increase in observed incidence. However, we noted no increasing attention to zoster in the popular media nor a concern among physicians about a projected increase in incidence. Thus, we doubt that the observed trend is due to greater awareness.

We did not collect data on underlying immunosuppressive conditions, which would have permitted us to examine the extent to which changes in herpes zoster incidence were related to changes in the prevalence of such diseases or therapies. Although it is unlikely this prevalence changed much in the five-year study period, it is conceivable that the proportion of respondents with such conditions varied randomly from year to year, which could have affected the results.

Finally, the response rate for the BRFSS has been uneven, hitting a low of 41 percent in 2000. If the incidence of varicella or herpes zoster differs among responders and non-responders, this could introduce some bias in the estimates of both the absolute incidence and of trends over time. However, a comparison of the 2000 BRFSS sample to the 2000 U.S. Census data for Massachusetts concluded that the BRFSS sample reflected its target population in most demographic and socioeconomic variables despite its low response rate [42]. Any differences in characteristics that might be relevant to herpes zoster incidence, such as race/ethnicity and proportion of households with children, were slight. The proportion of households with children in the sample varied little between 2000 and 2003, from a low of 37.1 percent to a high of 38.2 percent. In 1999, it was 33.7 percent, but given that the effect of living with children is thought to be protective, this difference would not explain our results.

## Conclusion

As varicella vaccine coverage in children increased, the incidence of varicella decreased and the occurrence of herpes zoster increased. If the observed increase is real, widespread vaccination of children is only one of several possible explanations.

Further research on herpes zoster is clearly warranted, particularly as a vaccine to prevent herpes zoster and its complications is likely to be available in the United States within the next few years [43-45]. A better understanding is needed of risk factors, the possible roles of internal (from subclinical reactivation) and external (from exposure to circulating VZV) boosting in preventing or delaying zoster, and temporal trends in incidence since mid-century. Additional methods for surveillance for herpes zoster should be considered to monitor the impact of widespread varicella vaccination of children as well as the impact of VZV vaccination of adults, should it become generally recommended.

## Competing interests

The author(s) declare that they have no competing interests.

## Authors' contributions

WKY helped design the questionnaire, interpreted the data, and drafted and revised the manuscript. DB helped design the questionnaire, oversaw implementation of the survey for several years, oversaw analysis of the data through 2002, interpreted the data, and critically reviewed all drafts of the manuscript, providing important insights. Recognizing the importance of carrying out the survey, SL secured funding to add the varicella and herpes zoster questions, and helped design the questionnaire, provided immunization data, and reviewed the manuscript. AJ contributed critical insights and raised questions in the interpretation of the data, provided comparative data and references, and critically reviewed all drafts of the manuscript. ZZ supported staff time for data analysis and himself re-analyzed the data through 2003; in addition, he helped interpret the data, ruling out certain artifactual explanations for the observations. KC analyzed the data through 2002 and provided much of the material for the methods section. JS funded the work, helped design the questionnaire, raised questions and contributed critical insights for the interpretation of the data, and critically reviewed the manuscript, supplying partial rewrites. All authors have approved the current version for publication.

## Pre-publication history

The pre-publication history for this paper can be accessed here:


